# Simultaneous intrathyroidal parathyroid adenomas and multifocal papillary thyroid carcinoma in a patient with kidney transplantation: a case report

**DOI:** 10.1186/s12882-019-1600-y

**Published:** 2019-11-09

**Authors:** Jun Yang, Jun Zhang, Jian-li Bi, Wan-wen Weng, Meng-jie Dong

**Affiliations:** Department of Nuclear Medicine, the First Affiliated Hospital, College of Medicine, Zhejiang University, 310003 Hangzhou, People’s Republic of China

**Keywords:** Intrathyroidal parathyroid adenomas, Hyperparathyroidism, Papillary thyroid carcinoma, Kidney transplantation

## Abstract

**Background:**

Persistent hyperparathyroidism after kidney transplantation has been associated with adverse outcomes. Parathyroidectomy is the definitive treatment approach, but the success of parathyroidectomy relies on the accurate preoperative localization of the culprit parathyroid lesions. Simultaneous intrathyroidal parathyroid adenomas and multifocal papillary thyroid carcinoma present important diagnostic challenges. Here, we describe a patient with kidney transplantation who underwent successful surgery after being evaluated with functional and structural imaging.

**Case presentation:**

A 53-year-old man presented with potentially malignant multifocal thyroid nodules by ultrasonography 2 years after kidney transplantation. The patient had hypercalcaemia and persistent hyperparathyroidism. Thyroid papillary carcinoma was confirmed in the left thyroid nodules by fine-needle aspiration biopsy. The right superior thyroid hypoechoic nodule was 1.2 cm in size and showed marked uptake of the tracer ^99m^TcO_4_-sestamibi during single-photon emission computed tomography/computed tomography (SPECT/CT); additionally, a cystic parathyroid lesion without tracer uptake was present behind the left superior pole of the thyroid. The histological examination demonstrated the coexistence of right intrathyroidal parathyroid adenomas, left cystic parathyroid nodular hyperplasia and multifocal papillary thyroid carcinoma. At the 6-month follow-up, the serum calcium levels were within the normal range, and the patient’s kidney function remained stable.

**Conclusions:**

Simultaneous intrathyroidal parathyroid adenomas and multifocal papillary thyroid carcinoma in a patient with kidney transplantation is a rare clinical scenario. Physicians must be aware that the combination of functional (SPECT/CT) and structural (ultrasonography) imaging is highly successful in diagnosing patients with coexistent intrathyroidal parathyroid adenomas and papillary thyroid carcinoma.

## Background

Kidney transplantation is an important strategy and the treatment of choice for patients with end-stage renal disease as this method will increase quality of life and improve patient survival [1]. Following kidney transplantation, parathyroid hyperplasia may slowly regress during recovery [2]. However, persistent post-transplant hyperparathyroidism (PT-HPT) is common because monoclonal parathyroid tissue either has a reduced response or lacks the typical response to physiologic regulatory mechanisms of parathyroid secretion and proliferation [3]. PT-HPT puts patients at high risk for bone fractures, mortality and renal graft loss [4, 5]. Surgical excision is considered as this method represents the only definitive cure. The precise preoperative localization of the abnormal parathyroid tissue, especially the identification of ectopic or supernumerary parathyroid tissue, is crucial to a successful surgery [6]. Currently, no consensus exists regarding the universally accepted imaging modalities for preoperative localization. Different imaging modalities have varying strengths and weaknesses depending on the clinical scenario [7]. Ultrasonography can help localize parathyroid disease and assess concomitant thyroid disease. However, ultrasonography has difficulty diagnosing intrathyroidal parathyroid adenoma (IPA) and particularly struggles in the differential diagnosis of thyroid nodules, IPA and papillary thyroid carcinoma [8]. ^99m^TcO^4^-sestamibi single-photon emission computed tomography/computed tomography (SPECT/CT) has a high sensitivity (84%) and positive predictive value (95%) and especially improves upon the detection rate of ultrasonography for ectopic tissue, which is likely to be missed by ultrasonography [9]. The combination of structural and functional imaging with ultrasonography and SPEC/CT may increase the localization accuracy and specificity to resolve this problem [10]. Here, we present a very rare case of simultaneous IPA, cystic parathyroid nodular hyperplasia and multifocal papillary thyroid carcinoma in a patient with kidney transplantation.

## Case presentation

**Fig. 1:**
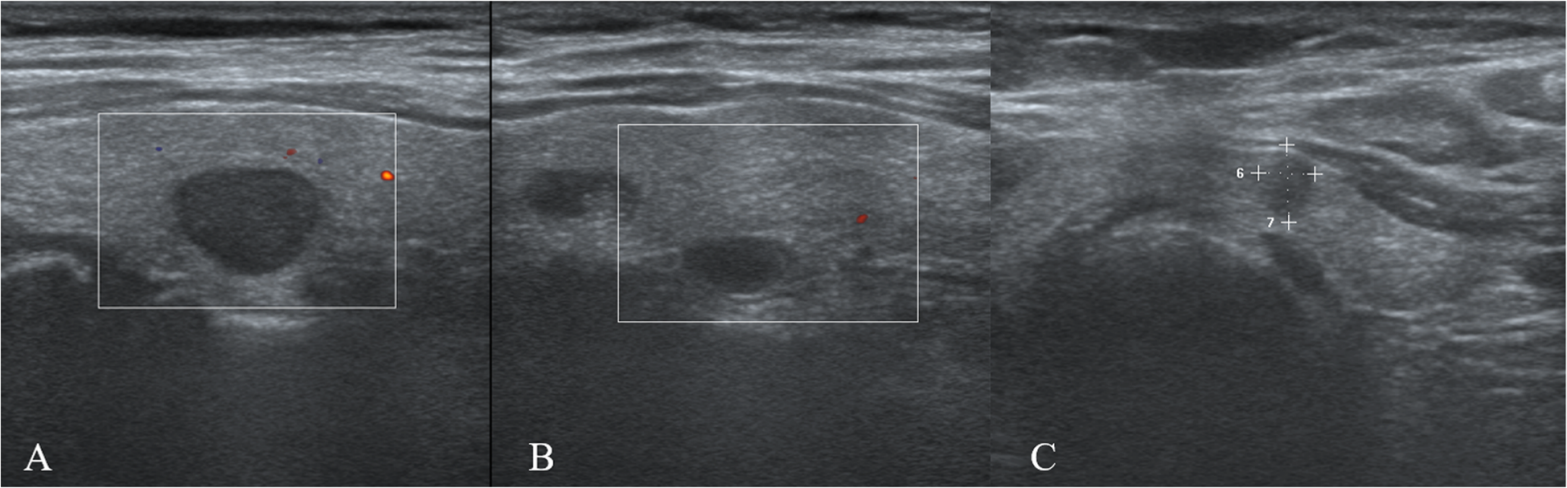
Ultrasonography image of hypoechoic nodules with well-defined margins located in the right inferior lobe of the thyroid gland (**a**). A typically cystic parathyroid adenoma with homogeneously hypoechoic and an elongated shape was located in posterior left superior thyroid (**b**). Ultrasonography showed a hypoechoic nodule with irregular margins with a taller-than-wide shape located in the left superior thyroid, which was confirmed as papillary carcinoma by FNAB (**c**)

**Fig. 2:**
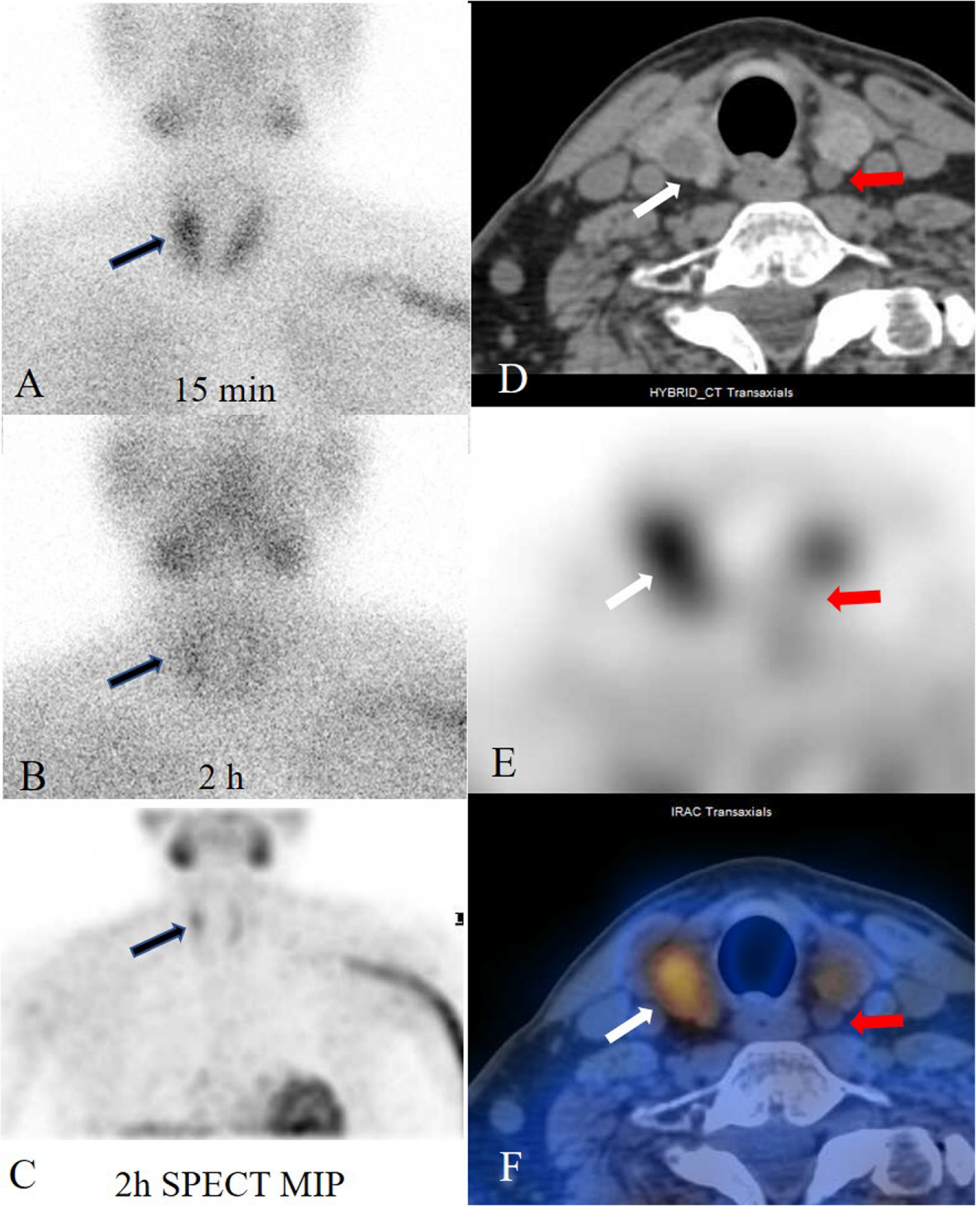
The ^99^mTcO4 MIBI dual-phase parathyroid scan detected elevated tracer uptake in the superior lobe of the thyroid in the early phase (**a**). A 2-h delayed image (**b**) showed mild retention of the tracer in the same region (black arrows). The SPECT/CT images at 2 h (**c**: MIP, maximum intensity projection; **d**: axial CT; **e**: SPECT; **f**: fused image) indicated that the focal tracer uptake was located in the right superior thyroid (white arrows). The lesion behind the superior left thyroid was a cystic nodule and had no radioactivity uptake (red arrows)

A 53-year-old man was admitted to our hospital in November 2018 because ultrasonography revealed potentially malignant multifocal nodules in the thyroid. The patient denied history of familial thyroid carcinoma and exposure of radiation. The patient’s previous medical history included peritoneal dialysis once per day since 2012 due to renal failure. He underwent kidney transplantation in our hospital on May 2016. The donor was the patient’s wife. Before surgery, secondary hyperparathyroidism was diagnosed as the patient had elevated intact-parathyroid hormone (iPTH 813.4 pg/mL, normal range 12–65 pg/mL), determined by an electrochemiluminescence immunoassay kit (Roche Diagnostics GmbH, Germany), and hypercalcemia. Ultrasonography revealed an orthotopic enlarged parathyroid nodule (1.1 cm) posterior to the left superior pole of the thyroid and multiple thyroid nodules. Kidney transplantation was successful with an immunosuppressive regimen (tacrolimus + mycophenolate + corticosteroids). The patient was conventionally administered to calcitriol and vitamin D. He had no signs and symptoms of hyperparathyroidism after kidney transplantation. During the 2-year follow-up period, his creatine, iPTH and serum calcium levels remained within 100–110 μmol/L (normal range 59–104 μmol/L), 131–156 pg/ mL, and 2.23–2.63 mmol/L (normal range 2.03–2.54 mmol/L), respectively. His physical examination was normal. A laboratory test showed that the blood creatinine was 126 μmol/L, blood urea nitrogen was 7.14 mmol/L (normal range 2.90–8.20 mmol/L), calcium was 2.80 mmol/L, phosphorus was 0.58 mmol/L (normal range 0.87–1.45 mmol/L), phosphatase was 124 U/L (normal range 40–150 U/L), iPTH was 220 pg/mL, and 25-hydroxyvitamin D was 71.7 nmol/L (normal range 12.3–107 nmol/L). His blood counts, thyroid function and tumour biological marker levels were normal. Ultrasonography showed multifocal hypoechoic nodules, and the largest nodule (1.2 cm) was located within the right thyroid lobe, with an enlarged cystic parathyroid gland posterior to the left superior pole of the thyroid (Fig. 1a, b). Thyroid papillary carcinoma was confirmed in the left thyroid nodules by fine-needle aspiration biopsy (FNAB) because the ultrasonographic findings suggested a malignancy (Fig. 1c). A ^99m^TcO_4_-sestamibi dual-phase parathyroid scan showed persistent uptake in the right inferior pole of the thyroid (Fig. 2a, b). Delayed-phase fused SPECT/CT images were acquired. The images demonstrated elevated tracer uptake in the nodule located in the right inferior thyroid and no focal retention in the cystic parathyroid tissue posterior to the left superior thyroid lobe (Fig. 2c, d, e, f).

**Fig. 3:**
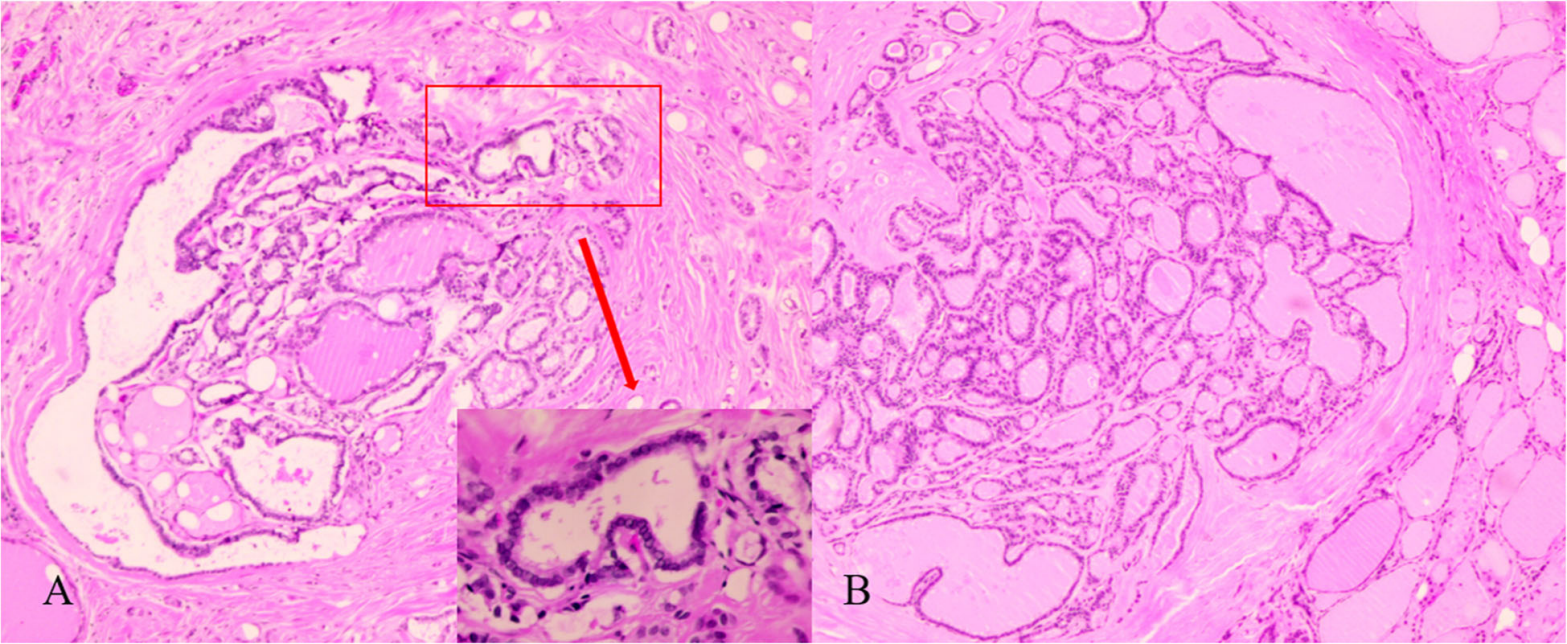
The histopathological images showed that multifocal papillary thyroid microcarcinoma was present in both thyroid lobes (**a**: left thyroid, haematoxylin and eosin (HE)-stained, magnification × 50; inset; × 200. **b**: right thyroid, HE-stained, magnification × 50)

**Fig. 4:**
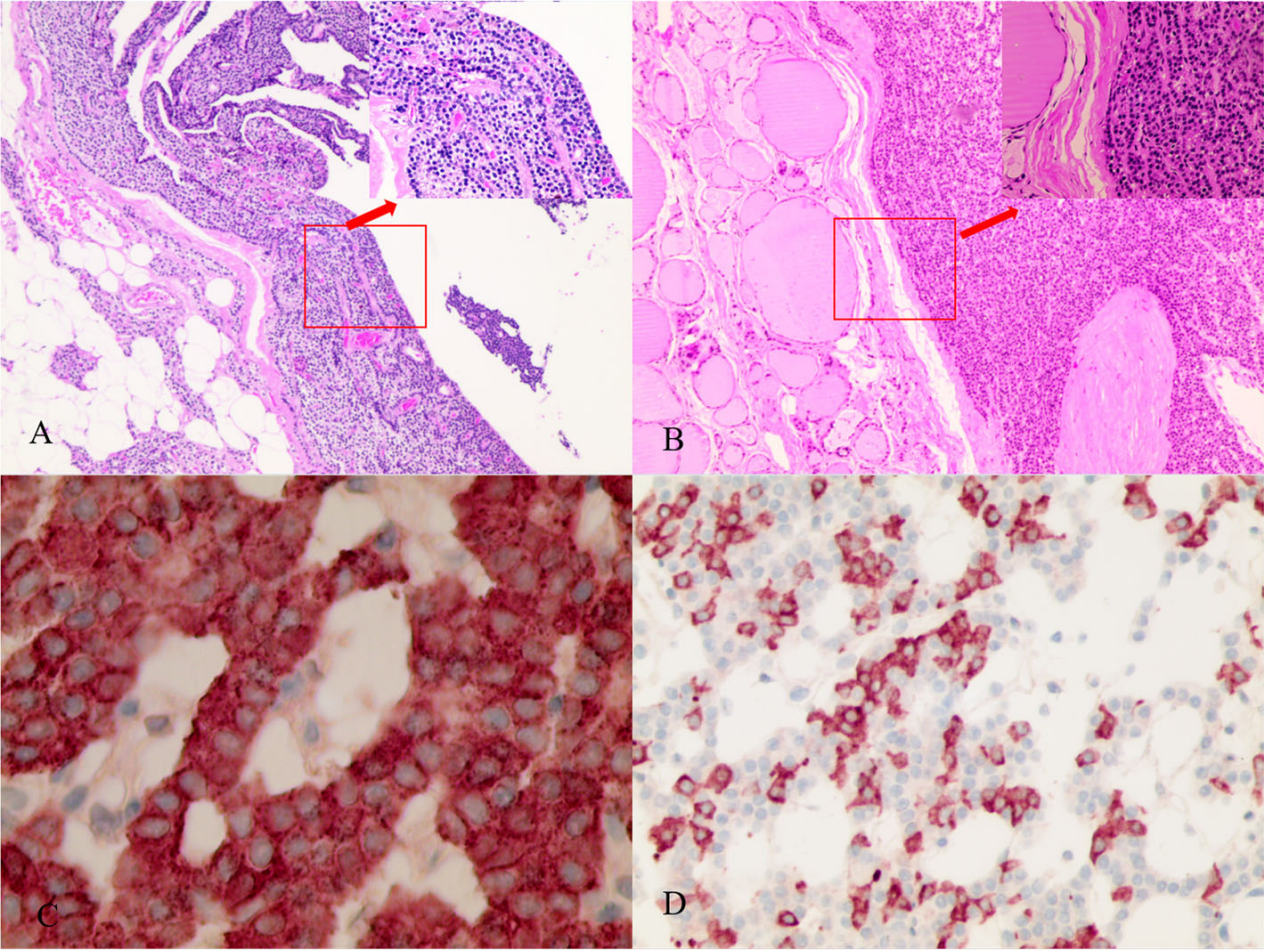
The left superior parathyroid adenoma showed cystic characteristics in the central region, and the peripheral region of the lesion was surrounded by parathyroid parenchyma (**a**: HE-stained, magnification × 50; inset; × 200). HE staining revealed that the encapsulated IPA was composed of chief cells surrounded by a rim of normal thyroid tissue, which can be seen in the upper right portion of the slide (**b**: HE-stained, magnification × 50; inset; × 200). Immunohistochemical staining of the IPA for chromogranin A (**c**) and synaptophysin (**d**) were positive

The patient underwent bilateral neck exploration. No other orthotopic abnormal parathyroid tissue was found. Left inferior cystic parathyroidectomy, thyroid lobectomy and central neck dissection were performed. The histopathological examination confirmed the diagnosis of multifocal papillary thyroid microcarcinoma and lymphatic metastasis (Fig. 3a, b). The left cystic lesion was confirmed to be parathyroid nodular hyperplasia, which consisted of a hyperplastic parathyroid surrounded by central cystic structures (Fig. 4a). The lesion located in the right inferior thyroid was diagnosed as IPA, which consisted of chief cells and was completely embedded within thyroid tissue (Fig. 4b). The immunohistochemical staining was positive for chromogranin A and synaptophysin in both parathyroid tissues (Fig. 4c, d). The serum calcium levels returned to normal, and iPTH decreased to 3.0 pg/mL 1 day after surgery. After surgery, calcium replacement with caltrate and alfacalcidol was administered. At 1-month postoperative, he was asymptomatic and calcium level was normal, he had been weaned off both agents. At the 6-month follow-up, the patient’s thyroid function achieved subclinical hyperthyroidism via an oral administration of L-euthyroxine (125 μg, once a day), his serum calcium was within the normal range, and his kidney function remained stable (Table 1).

**Table 1:** Results of laboratory values during the surgery and 6-month follow-up

Parameters	Reference range	Before kidney transplantation	After kidney transplantation	After parathyroidectomy	6-month follow-up
Date	NA	May 2016	Nov 2018	Dec 2018	May 2019
Albumin	35.0–55.0 (g/L)	40.8	44.7	41.0	45.6
Alkaline phosphates	40.0–150.0 (U/L)	107	124	93	83
Creatinine	59–104 (μmol/L)	**1317**	**126**	129	100
Total Calcium	2.03–2.54 (mmol/L)	2.44	**2.80**	2.25	2.33
Phosphorus	0.87–1.45 (mmol/L)	1.04	**0.58**	1.06	**0.86**
Thyroid stimulating hormone	0.38–4.34 (mIU/L)	1.23	3.22	NA	**0.063**
Intact-parathyroid hormone	12.0–65.0 (pg/mL)	**813.4**	**220**	**3.0**	84
25-hydroxyvitamin D	12.3–107 (nmol/L)	53.8	71.7	NA	67.8

Bolded values are out of the reference range; *NA* Not available

## Discussion and conclusions

Our report presents multiple challenges with respect to identifying the unique imaging features that clinicians should be aware of while managing patients with simultaneous hyperparathyroidism and thyroid nodules, especially patients with kidney transplantation. PT-HPT can be observed in some patients with successful kidney transplantation. PT-HPT occurs in 45 and 20% of kidney transplantation recipients after 2 years and 5 years, respectively [1]. The main cause of PT-HPT is enlarged and autonomous adenomas with dysregulated metabolic bone mineral parameters, even when the renal function of the allograft recovers to normal [3]. Studies have reported that PT-HPT is associated with an elevated risk of fractures, cardiovascular disease, allograft loss (5%) and mortality (4%) [4, 5, 11, 12]. Treatment for PT-HPT should be individualized, and parathyroidectomy should be considered.

Four orthotopic parathyroid glands are usually located behind the thyroid and originate from the third and fourth pharyngeal pouch. For successful parathyroidectomy, an accurate preoperative localization of the culprit parathyroid tissues is critical. Ectopic parathyroid adenomas occur in approximately 20 and 66% of patients with primary hyperparathyroidism and patients who undergo reoperation, respectively [6]. IPA is rarely observed in certain locations in the parathyroid. The incidence of IPA has been reported to be 1.3–6.7% [13, 14], but the literature has reported that the incidence of parathyroid adenomas located completely within the thyroid is less than 1%, and the most common location is inferior to the thyroid [15]. Some scientists speculate that the primordium of the parathyroid gland was trapped and migrated within the thyroid, thus forming as an intrathyroidal parathyroid gland. Ultrasonography and ^99m^TcO^4^-sestamibi SPEC/CT are the most common imaging modalities used to locate abnormal parathyroid glands. Ultrasonography can facilitate an excellent assessment of parathyroid adenomas (sensitivity: 76%, positive predictive value: 93%), but ultrasonography cannot detect ectopic sites, such as mediastinal, retroclavicular and retrooesophageal sites [16]. Ultrasonography is also relatively insensitive in detecting concurrent thyroid nodules and has challenges in identifying IPA [17]. Thyroid nodules occur in the endemic areas of up to 68% of all patients [18]. In total, 3% of patients have occult papillary carcinoma, and the incidence increases with age [19]. The incidence of thyroid papillary carcinoma in kidney transplant recipients is low, some studies evaluated that the incidence of thyroid cancer was 0.22–2.3% [20, 21]. Although the hyperechoic line on the ventral surface of the nodule is an important clue for diagnosing IPA, our patient had no characteristic imaging features [22]. Therefore, further evaluation was required with ^99m^TcO^4^-sestamibi SPEC/CT, which has high sensitivity (84%) and positive predictive value (95%) and improves upon the detection rate of ultrasonography for ectopic lesions [9]. In our case, ultrasonography only found one cystic parathyroid nodule without tracer uptake, and the left thyroid nodule was confirmed as papillary carcinoma by FNAB. Focal uptake in the right superior thyroid nodule on SPECT/CT is consistent with IPA due to persistent hyperparathyroidism after kidney transplantation. Some studies report that the combination of ultrasonography and ^99m^TcO^4^-sestamibi SPECT can accurately locate IPA [8–10, 23–27].

When IPA and multifocal papillary thyroid carcinoma are diagnosed in patients with kidney transplantation, no consensus guidelines exist for the best practices. Therefore, the decisions depend on the individual patient characteristics and the surgeons’ preferences, and the treatment would maintain normal calcium levels and renal function.

To the best of our knowledge, this is the first report of simultaneous IPA and multifocal papillary thyroid carcinoma in a patient with kidney transplantation. Although the precise localization and differential diagnosis of these conditions are challenging, the combination of ultrasonography, SPECT/CT and careful interpretation could help clinicians confirm the presence of IPA and papillary thyroid carcinoma.

## Data Availability

All datasets and figures supporting our findings are contained within the manuscript.

## Abbreviations

IPA: Intrathyroidal parathyroid adenoma
PTH: Parathyroid hormone
PT-HPT: Persistent post-transplant hyperparathyroidism
SPECT/CT: Single-photon emission computed tomography/computed tomography
